# Tensile Constitutive Model of Engineered Cementitious Composites Reinforced by High-Strength Steel Wire Mesh

**DOI:** 10.3390/ma17194709

**Published:** 2024-09-25

**Authors:** Jing Li, Ruiyuan Gao, Ang Wang, Ke Li, Di Wu, Hao Li, Yuxuan Li

**Affiliations:** 1 Civil Engineering and Construction Center, Huanghe Science and Technology University, Zhengzhou 450061, China; ljhm202305052@hhstu.edu.cn (J.L.); gry@hhstu.edu.cn (R.G.); wangang@hhstu.edu.cn (A.W.); 2 School of Civil Engineering, Zhengzhou University, Zhengzhou 450001, China; 3 College of Construction Engineering, Jilin University, Changchun 130012, China; dwu21@mails.jlu.edu.cn; 4 Xi’an Noble Metal Material Co., Ltd., Xi’an 710018, China; lihao3547@gmail.com; 5 School of Civil Engineering, Southwest Jiaotong University, Chengdu 611756, China; likeirwin@163.com

**Keywords:** engineered cementitious composites, high-strength stainless steel wire mesh, constitutive model

## Abstract

The presentation of a constitutive model could help researchers to predict the mechanical behavior of a material, which also contributes to the further generalization of the material. This paper is to explore the tensile constitutive model of engineered cementitious composites (ECCs) reinforced by high-strength steel wire mesh based on experiments and numerical simulations. DIANA was used to simulate the tensile process of the specimens, and experiments were carried out to validate the numerical model. The effect of the ECCs’ tensile strength, reinforcement ratio and specimen size were considered during the specimen design process. The results showed that most of the errors of the simulated values compared to the experimental results were within 5%, which proved that the numerical model was quite accurate. The proposed constitutive model revealed the different roles played by ECCs and high-strength steel wires at different stress stages, and the calculation results were in high agreement with the simulation results, indicating the effectiveness of the constitutive model. The study in this paper could provide an important reference for the popularization and application of ECCs reinforced by high-strength steel wire mesh.

## 1. Introduction

Concrete is one of the most widely used materials in the civil engineering field, but its disadvantages such as easy cracking, low tensile strength and poor toughness make it not reliable enough in some scenarios [1,2,3,4]. Engineered cementitious composites (ECCs) are micro-mechanically designed fiber-reinforced composites, which typically have greater toughness and better durability compared to conventional concrete, showing a broad application prospect [5,6,7].

There is a large number of studies focusing on the mechanical properties of ECCs, which is conducive to the further promotion of this material. Li et al. investigated the tensile properties of ECCs through uniaxial tensile tests. The results showed that the ultimate tensile strain of ECCs was more than 4%, and the crack width under the ultimate tensile strain was limited to less than 100 μm, which proved that ECCs had a better crack control ability [8]. Yu et al. suggested that due to the presence of fibers, the shear damage of the matrix during the compression of ECCs is greatly limited, and the specimens can bear significant compressive deformation and maintain integrity [9]. In practice, different types of ECC materials often have different mechanical properties due to differences in raw materials and mixing ratios. Shi et al. investigated the effect of different PVA fiber dosages on the bending strength of ECCs. The results showed that the bending strength increased when the fiber dosage was increased from 1% to 1.5%, while the strength started to decrease when the fiber dosage was increased to 2% [10]. Li et al. pointed out that the uniaxial compressive strength of ECCs increased with a decrease in the water/cement ratio, but if the water/cement ratio was too small, the compressive toughness of ECCs reduced. Finally, a reasonable water/cement ratio should be 0.17 [11]. In fact, regardless of the constituent materials and formulations, ECCs have strong advantages over traditional concrete, especially in terms of durability and toughness. So, it is a material worthy of further promotion.

However, studies have shown that some properties of ECCs are still limited [12]. For example, when the composites are exposed to moderately elevated temperatures (100–200 °C), the changes in strength and stiffness were quite obvious [13]. On the other hand, residual stiffness, which refers to the ability of a material to maintain its load-carrying capacity after initial cracking, is also lacking for ECCs [14,15]. Therefore, the use of ECCs in combination with other high-performance materials is essential. Fischer et al. conducted uniaxial tensile tests on rebar-reinforced ECCs, and the results showed that the crack distribution of reinforced ECC specimens was more uniform, the crack width was reduced, and the deformation control capacity was improved [16]. On the other hand, the use of rebar would prevent the advantages of ECCs’ high toughness from being fully utilized. Mihashi et al. performed uniaxial tensile tests on fiber-mesh-reinforced ECCs, and the results showed the axial stiffness and load capacity of the reinforced ECCs were significantly improved [17]. But the adoption of fiber mesh often means higher economic costs. As a popular reinforcing material, high-strength steel wire is now mainly used in combination with penetrating polymeric mortars to strengthen building structures [18]. Considering its high tensile strength and corrosion resistance as well as lower economic cost, the use of high-strength steel wire mesh to reinforce ECCs is proposed in this paper.

There have been some studies focusing on the mechanical properties of ECCs reinforced with high-strength steel wire mesh [19,20]. However, the promotion of a new material requires an in-depth understanding of its stress–strain relationships [21,22,23]. Experiments and numerical simulations are usually performed to obtain the corresponding constitutive models [24,25,26,27,28,29,30]. Wang et al. obtained a constitutive model for high-strength structural steels based on experiments [31]. Li et al. obtained a constitutive model of claystone based on numerical simulations [32]. Experiments are the simplest and most effective approach to obtain the stress–strain relationship of materials. However, due to the limitation of experiment conditions, it is sometimes very difficult to obtain a complete stress–strain curve. Numerical simulation methods often have to simplify the boundary conditions and material properties during the simulation process, which affects the results to some extent. In this paper, experiments and numerical simulations were used in combination to explore the tensile constitutive model of engineered cementitious composites reinforced by high-strength steel wire mesh.

To summarize, this paper proposed the use of high-strength steel wire mesh to enhance ECCs and explored the corresponding tensile constitutive model. DIANA was used to simulate the tensile process of the specimens, and experiments were carried out to verify the rationality. The effect of ECCs’ tensile strength, reinforcement ratio and specimen size were considered during the specimen design process. The study in this paper could provide an important reference for the popularization and application of reinforced ECC materials.

## 2. Materials and Methods

### 2.1. Geometric Modeling and Material Properties

**Figure 1:**
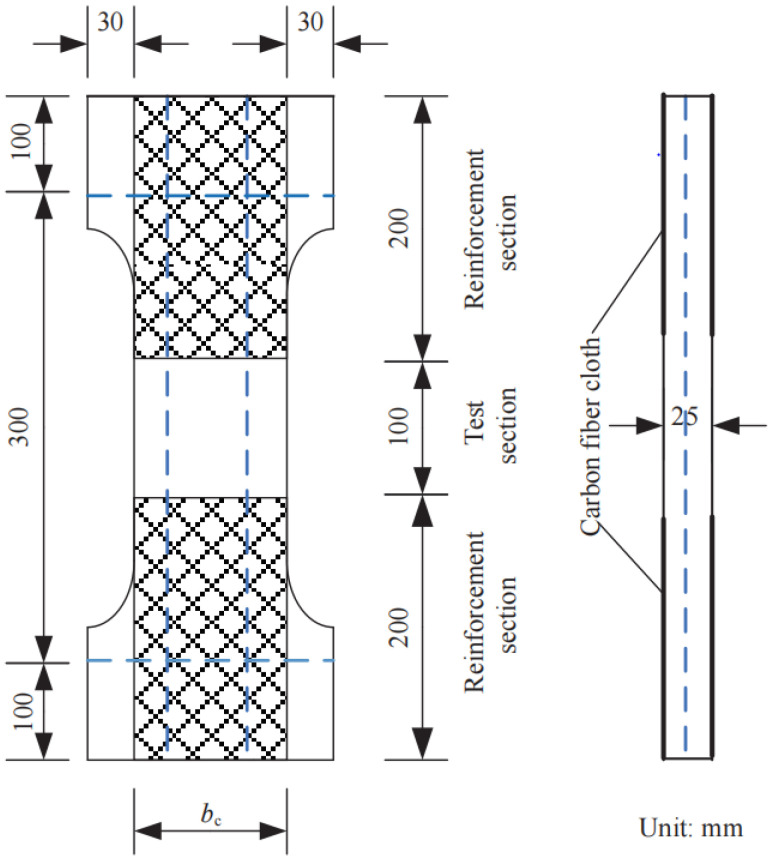
Schematic diagram of the specimen.

Considering the effect of ECCs’ tensile strength, reinforcement ratio and the size of the specimens, a total of 9 sets of specimens were prepared and the details are shown in Table 1**.** Each set has 3 specimens. To match the dimensions of the test machine and fit the situation in real applications, the geometric design of the specimens is shown in Figure 1. The ECCs were manufactured using ordinary Portland cement (PO 42.5), fly ash, silica fume, silica sand, polyvinyl alcohol (PVA) fibers, water, superplasticizer and thickener. With reference to a previous study, the proportions of ECCs for TC and TD groups are shown in Table 2 [33]. 

Two types of ECCs and high-strength steel wires were the main materials that made up the specimens. Based on previous studies [34], the constitutive model of the ECCs was as follows:

(1) $$\sigma_{e}=E_{e}\varepsilon_{e}\;\varepsilon_{e}\leq \varepsilon_{e,\mathrm{tc}}$$

(2) $$\sigma_{e}=(0.31\frac{\varepsilon_{e}}{\varepsilon_{e,tp}}+0.69)\sigma_{e,tp}\;\varepsilon_{e,\mathrm{tc}}\leq \varepsilon_{e}\leq \varepsilon_{e,\mathrm{tp}}$$

(3) $$\sigma_{e}=\sigma_{e,tp}-\sigma_{e,\mathrm{tp}}\frac{\varepsilon_{e}-\varepsilon_{e,tp}}{\varepsilon_{e,tu}-\varepsilon_{e,tp}}\;\varepsilon_{e,\mathrm{tp}}\leq \varepsilon_{e}\leq \varepsilon_{e,\mathrm{tu}}$$

where *σ_e_* is the tensile stress of the ECCs; *E_e_* is the elasticity modulus of the ECCs; *σ_e,tp_* is the tensile strength of the ECCs; and *ε_e_*, *ε_e,tc_*, *ε_e,tp_* and *ε_e,tu_* are the tensile strain, cracking strain, the tensile strain corresponding to the tensile strength and the ultimate tensile strain of the ECCs, respectively. The relevant parameters of the ECCs are shown in Table 3.

For the high-strength steel wires, the constitutive model can be fitted based on cubic polynomials [35]:

(4) $$\sigma_{s}=[a\frac{\varepsilon_{s}}{\varepsilon_{su}}+(3-2a){\left(\frac{\varepsilon_{s}}{\varepsilon_{su}}\right)}^{2}+(a-2){\left(\frac{\varepsilon_{s}}{\varepsilon_{su}}\right)}^{3}]\sigma_{su}$$

where *σ_s_*, *ε_s_*, *σ_su_*, *ε_su_*, are tensile stress, tensile strain, ultimate tensile stress (1568.41 Mpa) and ultimate tensile strain (0.0299) of the high-strength steel wires. a is a constant related to the diameter of the steel wires. For the high-strength steel wire with a diameter of 2.4 mm selected for this paper, a was determined to be 3.33.

### 2.2. Unit Type and Mesh Size

HX24L was adopted for ECCs in the present study. The HX24L element is an eight-node isoparametric solid brick element. It is based on linear interpolation and Gauss integration. As for the high-strength steel wires, embedded rebar units were selected and bonded slip reinforcement built into the DIANA 10.5 software was adopted. 

In finite element numerical simulations, the determination of the mesh size has a significant impact on the final results. With reference to previous studies [36], the choice of mesh size could be related to the surface area of the component, and the following equations are given:

Coarse mesh size = 0.049 (Overall surface area of the component)^1/2^ (5)

Fine mesh size = 0.0245 (Overall surface area of the component)^1/2^ (6)

To ensure the accuracy of the results while controlling the computational cost, the mesh size was finally taken as 10 mm. 

### 2.3. Interactions between Different Components

For the interaction between ECCs and high-strength steel wires, a local bond–slip model was applied. The corresponding formulas are as follows:

(7) $$\tau =\left\{\begin{array}{c} \tau_{u,l}{\left(\frac{s}{s_{o,l}}\right)}^{0.28},\;s\leq s_{o,l}\\ \tau_{u,l}(1-0.25(\frac{s}{s_{o,l}}-1)),\;s_{o,l}\leq s\leq s_{r,l}\\ \tau_{u,l}(1-0.25(\frac{s_{r,l}}{s_{o,l}}-1)),\;s>s_{r,l} \end{array}\right.$$

(8) $$\tau_{u,l}=5.12f_{t}(-0.125d/d_{2.4}+1.15)$$

(9) $$s_{o,l}=0.066d+0.253$$

(10) $$s_{r,l}=0.247d+0.511$$

where *τ_u_*_,*l*_ is the maximum local bonding stress of steel wires in ECCs; s_0,l_ is the slip corresponding to *τ_u_*_,*l*_; *s_r_*_,*l*_ is the slip corresponding to the end of the local bonding stress drop section; ft is the tensile strength of ECCs; d is the diameter of steel wire; *d*_2.4_ = 2.4 mm. 

### 2.4. Boundary Conditions and Loading Procedure

**Figure 2:**
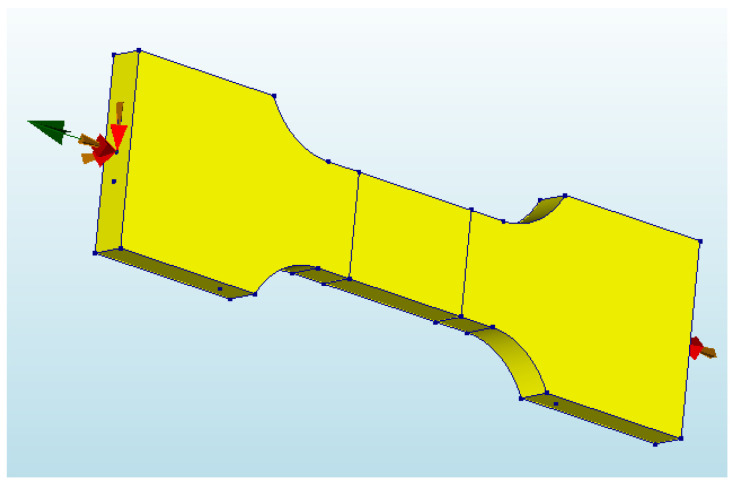
Schematic diagram of the model boundary and loading procedure.

The model used in this paper coupled the geometrical surfaces of the two ends at two separate points and imposed a fully fixed constraint on the coupling point. Subsequently, the coupling point at the tension end was moved to generate the load. The type of degrees of freedom at the loading point was defined as translational and the deformation in the x-direction was specified. A displacement-based graded loading procedure was set up with 0.2 mm per level. The model boundaries and loading procedure are shown in Figure 2.

### 2.5. Validation of the Numerical Model

**Figure 3:**
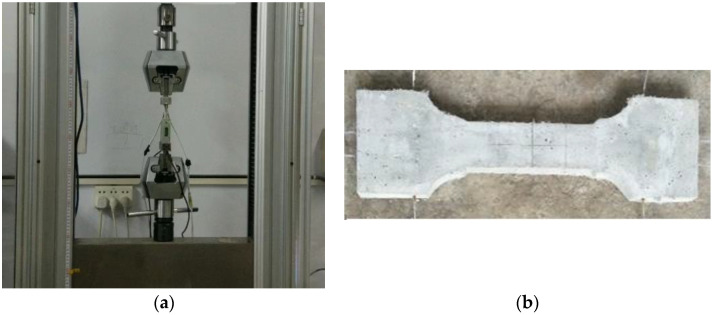
Experimental schematic: (**a**) loading device; (**b**) specimen.

Experiments were carried out to verify the numerical simulations with the above-mentioned geometrical properties, material properties, boundary conditions and loading procedures (Figure 3). The tensile process was realized based on an electro-hydraulic servo universal testing machine CMT5105, which is made by Zhengzhou Tianyu Technology Development Company. The company is loacated in Zhengzhou, Henan Povince, China. Strain gauges were pasted on the front and back sides of the specimen, and displacement gauges with a range of 30 mm were arranged on both sides of the specimen test section. The specific experimental procedures are as follows: (1) Switch on the testing machine and preheat it for 15 min; (2) select suitable tensile fixtures according to the size of the specimen; (3) switch on the servo oil pump to raise the working table; (4) clamp one end of the specimen; (4) adjust the position of the beam to clamp the other end of the specimen; (5) zero the strain value; (6) carry out the test at a preset loading rate; (7) stop the test and save the data after the specimen breaks. It should be noted that the test procedure is carried out in a defined working environment, i.e., the room temperature was 10–35 °C and the relative humidity was less than 80 percent.

### 2.6. Validation of the Constitutive Model

When the derivation of the constitutive model was completed, additional specimens needed to be designed to verify the model. Without changing the specimen dimensions, the parameters of the TC and TD group specimens were changed, and the parameters of the additional specimens are shown in Table 4 (LA group) and Table 5 (LB group).

## 3. Results

### 3.1. Simulation and Experimental Results

A high-quality numerical simulation process should reproduce the experimental process as realistically as possible. Taking specimen TD12 as an example, the entire loading process of the numerical simulation and experiment was compared.

**Figure 4:**
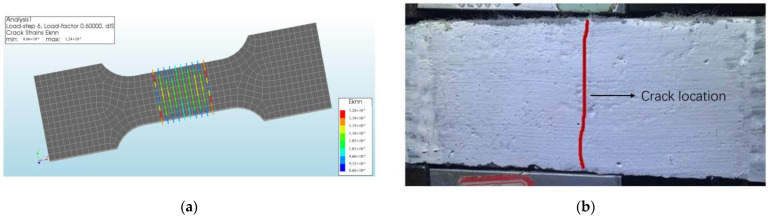
Simulated and experimental result at specimen cracking: (**a**) simulated strain cloud diagram; (**b**) experimental specimen status.

The specimen cracked when the tensile stress reached 2.09 MPa. As shown in Figure 4, the crack strain in the middle of the specimen could explain the cracking of the specimen in the middle during the experiment.

**Figure 5:**
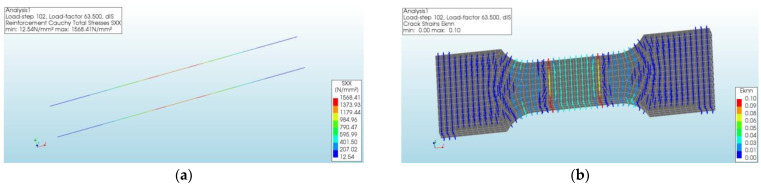
Cloud diagram of the ultimate load state of the specimen: (**a**) stress cloud of longitudinal high-strength steel wires; (**b**) strain diagram of the specimen.

When the tensile stress reached 7.11 MPa, the specimen reached the ultimate load state (Figure 5). The longitudinal high-strength steel wire also reached the ultimate stress of 1568.41 MPa. And the maximum strain was located at both ends of the test section.

**Figure 6:**
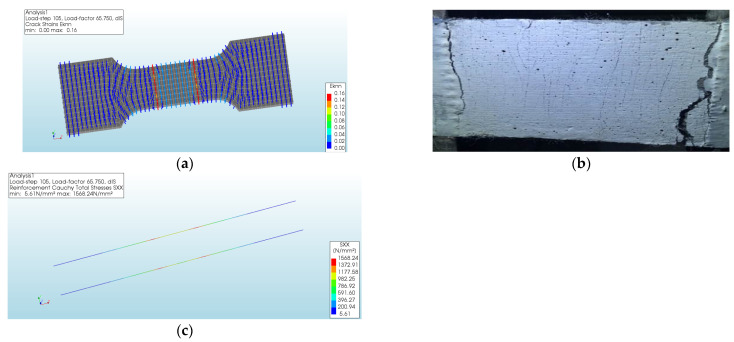
Simulated and experimental result at specimen destruction: (**a**) simulated strain cloud diagram of the specimen; (**b**) experimental specimen status; (**c**) simulated strain cloud diagram of the high-strength steel wires.

Subsequently, the tensile stress of the specimen began to decline. The maximum crack strain was located at both ends of the test section of the specimen, as shown in Figure 6, indicating that the main crack was formed here, which fit well with the experimental results. On the other hand, the stress changes of the longitudinal high-strength steel wires were quite small, indicating that the decrease in tensile stress of the specimen was mainly caused by ECCs.

Table 6 demonstrated the cracking loads and ultimate loads obtained from simulations and experiments. The error was the ratio of the difference between the simulated and experimental values to the experimental value. The results showed that most of the errors of the simulated values compared to the experimental results were within 5%, which proved that the numerical model was quite accurate. On the other hand, most of the cracking stress and cracking strain of the specimens obtained from the simulation had smaller errors, which indicated that the numerical model was able to better reproduce the stress state at the elastic stage of the specimens. After the cracking of the specimens, the stress state of the specimens became more complicated, which led to a larger error in the simulation results.

### 3.2. Derivation of the Tensile Constitutive Model

(1) Elasticity Stage

In the elastic stage, the external load was shared by the high-strength steel wire and ECCs, and there is no slip between them. The constitutive model can be described as follows:

(11) $$\sigma_{\mathrm{se}}=(E_{e}+E_{s}\frac{A_{s}}{A_{se}})\varepsilon_{se}\;\varepsilon_{\mathrm{se}}\leq \varepsilon_{se,tc}$$

where E_e_ and E_s_ are the elasticity modulus of ECCs and high-strength steel wires; A_se_ is the cross-sectional area of ECCs reinforced by high-strength steel wire mesh; A_s_ is the cross-sectional area of high-strength steel wires; *σ_se_* and *ε_se_* are the tensile stress and tensile strain of ECCs reinforced by high-strength steel wire mesh.

(2) Plasticity Stage

Before the ultimate load state, the external load was also shared by both ECCs and high-strength steel wires, and the slip between them should be considered. Accordingly, the following formula could be proposed:

(12) $$\sigma_{\mathrm{se}}=\gamma \sigma_{s}\rho_{s}+\sigma_{e}$$

Substitute Formulas (2) and (4) into Formula (6), based on the experimental results, making *ε_su_* = *ε_se,tp_* = *ε_e,tp_*, then the constitutive model can be described as follows:

(13) $$\sigma_{\mathrm{se}}=\gamma [a\frac{\varepsilon_{se}}{\varepsilon_{se,\mathrm{tp}}}+(3-2a){\left(\frac{\varepsilon_{se}}{\varepsilon_{se,\mathrm{tp}}}\right)}^{2}+(a-2){\left(\frac{\varepsilon_{se}}{\varepsilon_{se,\mathrm{tp}}}\right)}^{3}]\sigma_{su}\frac{A_{s}}{A_{se}}+(0.31\frac{\varepsilon_{se}}{\varepsilon_{se,\mathrm{tp}}}+0.69)\sigma_{e,tp}\;\varepsilon_{se,tc}\leq \varepsilon_{\mathrm{se}}\leq \varepsilon_{se,tp}$$

where *σ_se_* is the tensile stress of ECCs reinforced by high-strength steel wire mesh; *σ_su_* is the tensile strength of the high-strength steel wires; *σ_e,tp_* is the tensile strength of ECCs; *ε_se_*, *ε_se,tc_* and *ε_se,tp_* are the tensile strain, cracking strain and strain corresponding to the tensile strength of ECCs reinforced by high-strength steel wire mesh; *γ* is the stress factor for high-strength steel wires (0.725); a is a is a constant related to the diameter of the steel wires. For the high-strength steel wire with a diameter of 2.4 mm selected for this paper, a was determined to be 3.33.

After the ultimate load state, since the decrease in tensile stress of the specimens was mainly caused by the ECCs, the following formula could be proposed:

(14) $$\sigma_{\mathrm{se}}=\gamma \sigma_{\mathrm{su}}+[\sigma_{e,tp}-\alpha \frac{\sigma_{e,tp}}{\varepsilon_{e,tu}-\varepsilon_{e,tp}}(\varepsilon_{se}-\varepsilon_{se,tp})]$$

Considering the formula as a linear function, it can be simplified as follows:

(15) $$y=A+k(x-\varepsilon_{se,tp})$$

(16) $$k=\frac{-\alpha \sigma_{e,tp}}{\varepsilon_{e,tu}-\varepsilon_{e,tp}}$$

Fitting the linear function using the simulation results of different specimens, the values of k and α can be obtained (Table 7). It can be seen that α is mainly related to the reinforcement ratio ρ. The expression for α can be obtained by further fitting as follows:

(17) α=0.692+223(ρ−0.0028)

The constitutive model can be described as follows:

(18) $$\sigma_{\mathrm{se}}=\gamma \sigma_{\mathrm{su}}+\{1-[0.692+223(\frac{A_{s}}{A_{\mathrm{se}}}-0.0028)]\frac{\varepsilon_{se}-\varepsilon_{se,tp}}{\varepsilon_{e,tu}-\varepsilon_{e,tp}}\}\sigma_{e,tp}\;\varepsilon_{se,\mathrm{tp}}\leq \varepsilon_{se}$$

where σ_se_ is the tensile stress of ECCs reinforced by high-strength steel wire mesh; σ_su_ is the tensile strength of the high-strength steel wires; σ_e,tp_ is the tensile strength of the ECCs; ε_se_ and ε_se,tp_ are the tensile strain and tensile strain corresponding to the tensile strength of the ECCs reinforced by high-strength steel wire mesh; ε_e,tp_ and ε_e,tu_ are the tensile strain corresponding to tensile strength and the ultimate tensile strain of the ECCs; γ is the stress factor for high-strength steel wires; A_se_ is the cross-sectional area of ECCs reinforced by high-strength steel wire mesh; A_s_ is the cross-sectional area of the high-strength steel wires.

(3) Fitting Results of the Constitutive Model

**Figure 7:**
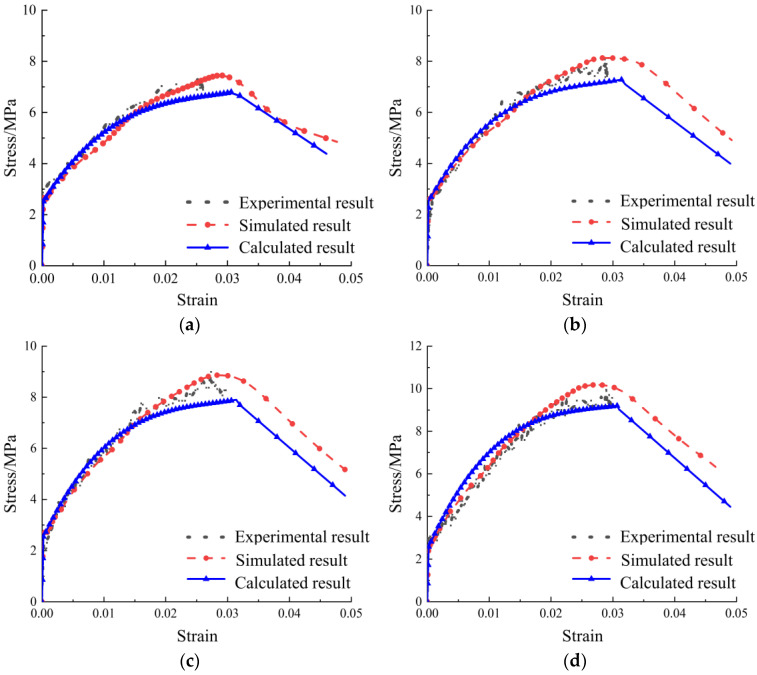

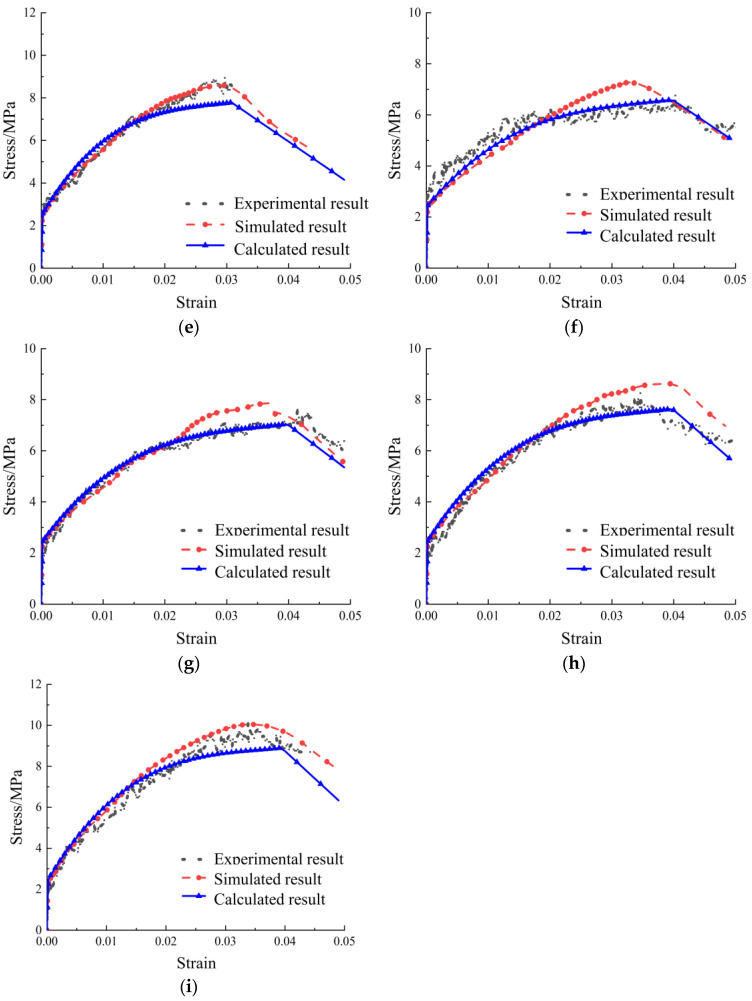
The comparative results of experiments, simulation and calculation: (**a**) TC12; (**b**) TC22; (**c**) TC32; (**d**) TC42; (**e**) TC52; (**f**) TD12; (**g**) TD22; (**h**) TD32; (**i**) TD42.

The calculated results of the constitutive model, the experimental results and the numerical simulation results were shown in Figure 7. It can be seen that the calculated results were in good agreement with the experimental and simulation results, which showed that the constitutive model was well-fitted.

### 3.3. Validation of the Tensile Constitutive Model

**Figure 8:**
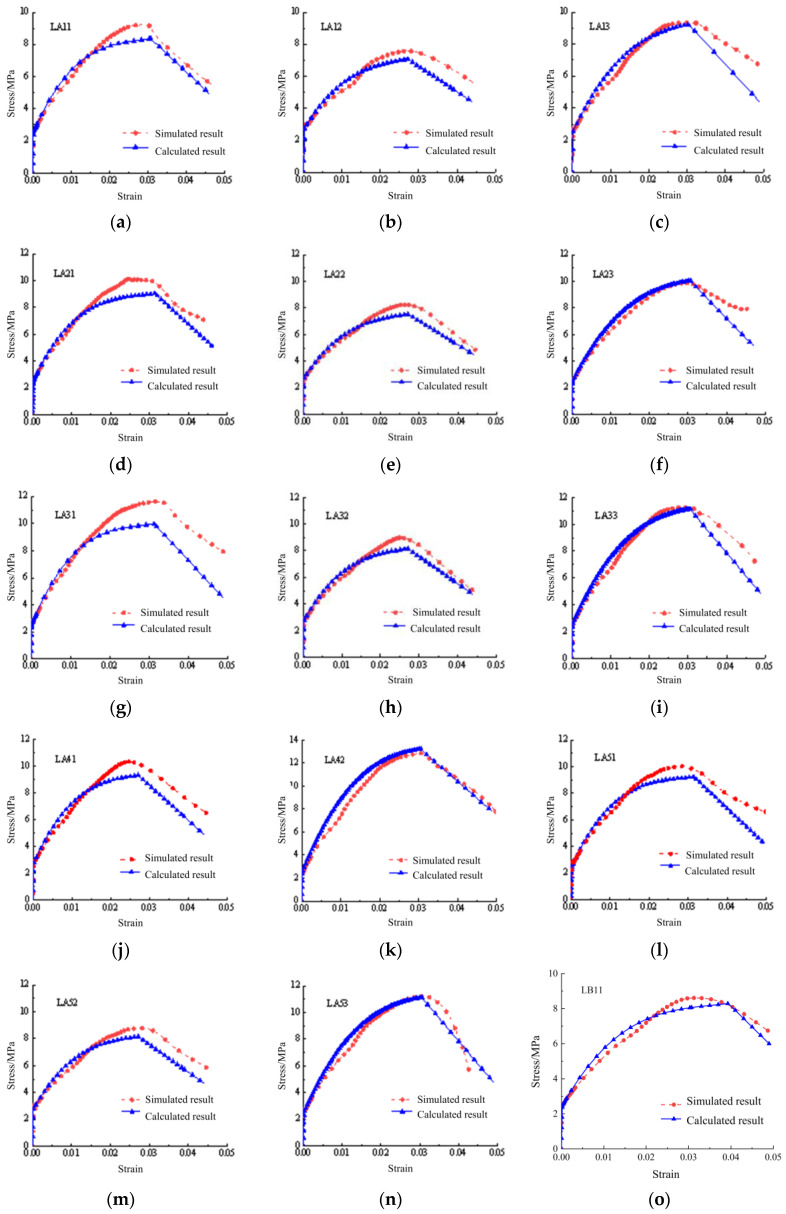

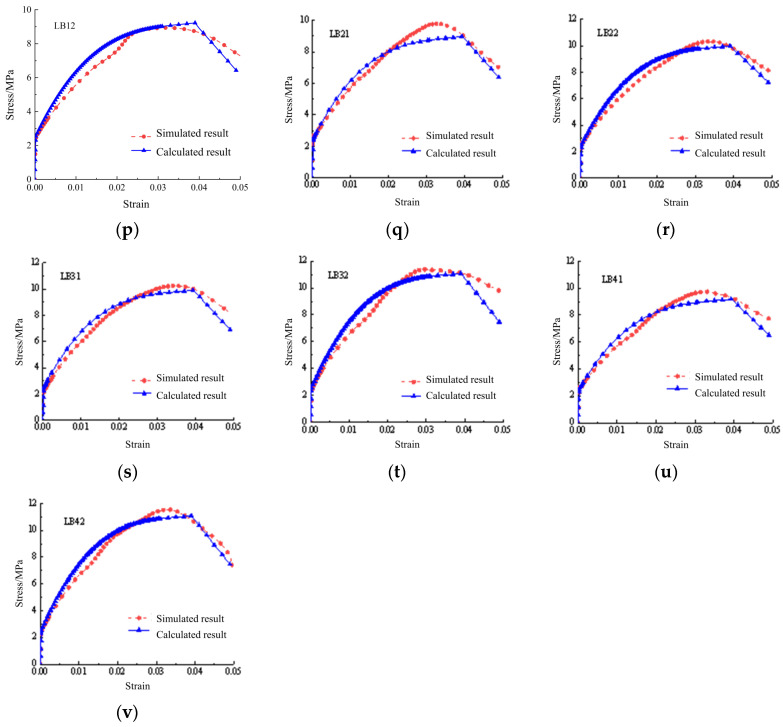
The comparative results of simulation and calculation: (**a**) LA11; (**b**) LA12; (**c**) LA13; (**d**) LA21; (**e**) LA22; (**f**) LA23; (**g**) LA31; (**h**) LA32; (**i**) LA33; (**j**) LA41; (**k**) LA42; (**l**) LA51; (**m**) LA52; (**n**) LA53; (**o**) LB11; (**p**) LB12; (**q**) LB21; (**r**) LB22; (**s**) LB31; (**t**) LB32; (**u**) LB41; (**v**) LB42.

Based on the validated numerical model and the constitutive model proposed in this paper, the specimens in Table 4 and Table 5 were fitted and calculated, respectively. The stress–strain curves are shown in Figure 8. Table 8 demonstrates the cracking loads and ultimate loads obtained from simulations and calculations. The results showed that the error of the calculation rarely exceeded 10%, which verified the reliability of the constitutive model.

## 4. Discussion

### 4.1. Reinforcement of ECCs

ECCs as a new type of building material have good physical and mechanical properties, a large peak tensile strain, good crack control ability and high toughness, which can give the engineering structure good mechanical properties, seismic performance and durability [37]. Numerous studies have shown that the advantages of ECCs can be further exploited by using ECCs in conjunction with reinforcing materials, and the degree of enhancement depends on the mechanical properties of the reinforcing materials as well as the adhesion between ECCs and the reinforcing materials [16]. Rebar is one of the most commonly used reinforcing materials. Experiments have shown that rebar-reinforced ECCs could provide improvement in load-carrying and crack control capacity [38]. On the other hand, the irrational use of conventional rebar may prevent the high ductility advantage of ECCs from being fully utilized. Fiber-reinforced polymer (FRP) is characterized by high strength, corrosion resistance and durability, so it is another common material used to reinforce ECCs. Studies have shown that the combination of FRP mesh and ECCs can further improve the toughness and crack control of the components [39]. However, the use of FRP usually implies higher economic costs. Considering the cost and the reinforcement effect, high-strength steel wire mesh with high tensile strength, good corrosion resistance and a low cost is used to reinforce ECCs. The results showed that the introduction of the high-strength steel wire mesh greatly increased the tensile strength of the specimens, and the high toughness and excellent crack control ability of ECCs were retained. The study in this paper provided a new material to enhance ECCs, which could help the further promotion of ECC materials.

### 4.2. Acquisition and Validation of Constitutive Models

There have been some studies focusing on the mechanical properties of ECCs reinforced with high-strength steel wire mesh. Li et al. explored the bending resistance of this material [19]. Zhu et al. investigated the bond–slip relationship at the interface between this material and concrete [20]. However, these studies were carried out based on specific specimens, which could not provide generalized knowledge for the generalization of ECCs reinforced with high-strength wire mesh. Therefore, the derivation of a constitutive model for this material is quite crucial.

A complete stress–strain curve is the basis for obtaining high-quality constitutive models [22]. Experiments are the most intuitive and accurate methods of obtaining stress–strain relationships, but some limitations are also evident. It is difficult to avoid errors in both the specimen preparation process and the experimental process, which would affect the results to some extent. On the other hand, due to the limitation of the experimental conditions, not every specimen could obtain its complete stress–strain curve [34]. For example, in this paper, most of the specimens were only able to obtain the stress–strain curves before the ultimate load state. Therefore, numerical simulations are usually introduced to obtain complete and accurate stress–strain curves. In this paper, numerical simulations and experiments were combined together to explore the stress–strain curves of the specimens. The results showed that most of the errors of the simulated values compared to the experimental results were within 5%, which proved the accuracy of the numerical model. On the other hand, the simulation results for the cracking stress of the specimens were more accurate compared to the ultimate stress, which indicated that the numerical model can better restore the stress state of the specimens in the elastic phase. The numerical model of the plastic phase needs to be further revised in future studies.

For the high-strength-steel-wire-mesh-reinforced ECCs, after obtaining their stress-strain curve, clarifying the contribution of each component at each stress stage is the key to further derive the constitutive model. In the elastic stage, the external load is shared by the high-strength steel wire mesh and ECCs, and there is no slip between them. The constitutive model can be described as a simple superposition of two constitutive models of ECCs and high-strength steel wires. In the plasticity stage, before the ultimate load state, the external load is also shared by both ECCs and high-strength steel wires, and the slip between them should be considered. So, the stress factor γ for high-strength steel wire mesh was introduced. After the ultimate load state, the stress reduction was mainly caused by ECCs, which is reflected in Formula (8).

The obtained constitutive model could describe the whole process of the specimens from the beginning of loading to their destruction well. The study in this paper could provide an important reference for the popularization and application of ECCs reinforced by high-strength steel wire mesh. In future studies, for the further promotion of the constitutive model, more specimens should be introduced to further verify the constitutive model.

## 5. Conclusions

In this paper, we proposed the use of high-strength steel wire mesh to reinforce ECCs and explored the corresponding tensile constitutive model. The study in this paper could help researchers to predict the mechanical behavior of the composite material, which also contributes to the further generalization of it. The following conclusions could be drawn:

(1) High-strength steel wire mesh could greatly increase the tensile strength of the specimens while retaining the high toughness and excellent crack control ability of ECCs.
(2) The combination of experiments and numerical simulations is an effective method to obtain complete stress–strain curves for the derivation of the tensile constitutive model of a new material.
(3) Clarifying the contribution of each component at each stress stage is the key to further derive the constitutive model of high-strength-steel-wire-mesh-reinforced ECCs.

## Figures and Tables

**Table 1:** Specimen design parameters.

Number	Test Section Width b_c_/mm	Distance between Steel Wires	Number of Steel Wires	Reinforcement Ratio	ECCs’ Tensile Strength
TC12	80	50	2	0.0028	3.53
TC22	70	40	2	0.0032	3.53
TC32	60	30	2	0.0037	3.53
TC42	47	20	2	0.0048	3.53
TC52	90	30	3	0.0037	3.53
TD12	80	50	2	0.0028	3.46
TD22	70	40	2	0.0032	3.46
TD32	60	30	2	0.0037	3.46
TD42	47	20	2	0.0048	3.46

**Table 2:** Mix proportions of ECCs.

Group	Cement	Sand	Fly Ash	Silica Fume	Water	PVA Fiber	Superplasticizer	Thickener
TC	1	0.3	4	0.08	1	0.02	0.06	0
TD	1	0.3	4	0.08	1	0.02	0.06	0.006

**Table 3:** Parameters of ECCs.

Type of ECCs	Cracking Stress/MPa	Cracking Strain	Tensile Strength/MPa	Strain Corresponding to Tensile Strength
Type 1	2.45	0.000204	3.53	0.0279
Type 2	2.13	0.000189	3.46	0.0297

**Table 4:** Parameters of LA group.

Number	Diameter of Steel Wires	Number of Steel Wires	Distance between Steel Wires (mm)	ECCs’ Tensile Strength
LA11	2.4	3	25	3.53
LA12	2.4	2	50	3.86
LA13	3.2	2	50	3.53
LA21	2.4	3	20	3.53
LA22	2.4	2	40	3.86
LA23	3.2	2	40	3.53
LA31	2.4	3	15	3.53
LA32	2.4	2	30	3.86
LA33	3.2	2	30	3.53
LA41	2.4	2	20	3.86
LA42	3.2	2	20	3.53
LA51	2.4	4	20	3.53
LA52	2.4	3	30	3.86
LA53	3.2	3	30	3.53

**Table 5:** Parameters of LB group.

Number	Diameter of Steel Wires	Number of Steel Wires	Distance between Steel Wires (mm)	ECCs’ Tensile Strength
LB11	2.4	3	25	3.46
LB12	3.2	2	50	3.46
LB21	2.4	3	20	3.46
LB22	3.2	2	40	3.46
LB31	2.4	3	15	3.46
LB32	3.2	2	30	3.46
LB41	2.4	4	20	3.46
LB42	3.2	2	30	3.46

**Table 6:** Simulated and experimental cracking loads and ultimate loads.

Specimen	Simulated Cracking Stress/MPa	Experimental Cracking Stress/MPa	Cracking Stress Error	Simulated Ultimate Stress/MPa	Experimental Ultimate Stress/MPa	Ultimate Stress Error
TC 12	2.21	2.20	0.45%	7.44	7.09	4.94%
TC 22	2.32	2.25	3.11%	8.13	7.94	2.39%
TC 32	2.40	2.41	−0.41%	8.86	8.77	1.03%
TC 42	2.49	2.56	−2.73%	10.18	10.65	−4.41%
TC 52	2.59	2.54	1.97%	8.57	8.82	−2.83%
TD 12	2.09	1.97	6.09%	7.11	6.41	10.92%
TD 22	2.13	1.99	7.04%	7.87	7.81	0.77%
TD 32	2.15	2.07	3.86%	8.62	8.62	0.00%
TD 42	2.21	2.19	0.91%	10.04	10.30	−2.52%

**Table 7:** The fitting results of α based on different specimens.

Specimen	TC12	TC22	TC32	TC42	TC52	TD12	TD22	TD32	TD42
k	−155	−198	−208	−249	−228	−141	−181	−195	−260
R^2^	0.9702	0.9979	0.9974	0.9952	0.9910	0.9857	0.9757	0.9794	0.9816
ρ	0.0028	0.0032	0.0037	0.0048	0.0037	0.0028	0.0032	0.0037	0.0048
α	0.692	0.883	0.930	1.110	1.020	0.629	0.808	0.870	1.160

**Table 8:** Simulated and calculated cracking loads and ultimate loads.

Specimen	Simulated Cracking Stress/MPa	Calculated Cracking Stress/MPa	Cracking Stress Error	Simulated Ultimate Stress/MPa	Calculated Ultimate Stress/MPa	Ultimate Stress Error
LA11	2.58	2.55	−1.16%	9.15	8.35	−8.74%
LA12	2.13	2.23	4.69%	7.57	7.08	−6.47%
LA13	2.62	2.54	−3.05%	9.34	9.24	−1.07%
LA21	2.52	2.56	1.59%	10.08	9.04	−10.32%
LA22	2.32	2.14	−7.76%	8.23	7.54	−8.38%
LA23	2.39	2.56	7.11%	9.85	10.06	2.13%
LA31	2.76	2.58	−6.52%	11.63	9.96	−14.36%
LA32	2.28	2.13	−6.58%	8.97	8.15	−9.14%
LA33	2.66	2.58	−3.01%	11.25	10.14	−9.87%
LA41	2.53	2.78	9.88%	10.35	9.34	−9.76%
LA42	2.63	2.61	−0.76%	12.85	13.25	3.11%
LA51	2.23	2.31	3.59%	10.03	9.24	−7.88%
LA52	2.22	2.15	−3.15%	8.79	8.15	−7.28%
LA53	2.24	2.29	2.23%	11.14	11.14	0.00%
LB11	2.27	2.21	−2.64%	8.60	8.28	−3.72%
LB12	2.22	2.26	1.80%	8.93	9.22	3.25%
LB21	2.16	2.22	2.78%	9.77	8.97	−8.19%
LB22	2.27	2.28	0.44%	10.32	9.97	−3.39%
LB31	2.12	2.24	5.66%	10.24	9.89	−3.42%
LB32	2.32	2.29	−1.29%	11.37	11.05	−2.81%
LB41	2.22	2.26	1.80%	9.73	9.17	−5.76%
LB42	2.22	2.29	3.15%	11.53	11.05	−4.16%

## Data Availability

The data presented in this study are available on request from the corresponding author (due to privacy).
